# Assessing the accuracy, reliability, and validity of menstrual cycle phase tracking equations in an applied sport setting

**DOI:** 10.1007/s00421-026-06211-y

**Published:** 2026-04-10

**Authors:** Casey Greenwalt, Paul Ansdell, Kevin Thomas, Kelly McNulty, Glyn Howatson, Amanda G. Mutuwa, Owen Munro, Kirsty M. Hicks

**Affiliations:** 1 Department of Sport, Exercise and Rehabilitation, Northumbria University, Newcastle-upon-Tyne, UK; 2 Performance, Medical & Innovation Department, Washington Spirit Soccer Club, Washington DC, USA; 3 Water Research Group, School of Environmental Sciences and Development, North West University, Potchefstroom, South Africa

**Keywords:** Female athlete, Menstrual cycle, Menstrual cycle tracking, Elite sport

## Abstract

**Purpose:**

To assess the accuracy, reliability, and validity of five mathematical equations across multiple cycles to predict menstrual cycle onset and date of ovulation in the applied environment.

**Methods:**

11 professional female athletes who were either naturally menstruating or intrauterine device (IUD) users tracked their menstrual cycles throughout the course of an entire national women’s soccer league (NWSL) season. Participants recorded the first and last dates of each menstrual cycle, which were used in mathematical equations to predict next bleed and ovulation date, which were validated by retrospective analysis and confirmed by ovulation tests from two consecutive cycles.

**Results:**

When predicting next bleed date, there was no difference in accuracy (MAE: 6 days), reliability (ICC3 = 0.60, 95% CI [0.53–0.68], *p* < 0.001) (CV: 24.3 to 25.1%), or prediction error (β = −0.20, SE = 0.16, t(335) = − 1.22, 95% CI [− 0.65 − 0.24]) between the equations, yet there was a significant, positive association between cycle length and prediction error (β = 1.10, SE = 0.03, t(335) = 31.54, 95% CI [0.94–1.13]). When predicting ovulation date, there was no significant difference in accuracy (MAE: 4), or reliability (ICC3 = 0.45, 95% CI [0.18–0.75], *p* < 0.001) (8.5–22.1%).

**Conclusion:**

No equation emerged as significantly better when predicting next bleed or next ovulation date, and all equations had relatively high variability and error rate. It is imperative that practitioners monitor menstrual characteristics and consider cycle variability when utilizing equations to predict menstrual cycle bleed and ovulation date.

## Introduction

In recent years, there has been a significant increase in female physiology research, particularly on the impact of the menstrual cycle on overall wellness, performance, and readiness to train in elite female athletes (Armour et al. 2020; Bruinvels et al. 2022; Engseth et al. 2025; Findlay et al. 2020; McNulty et al. 2024). In addition to reflecting the status of reproductive health, variability in menstrual cycle characteristics, such as menstrual cycle length, ovulation occurrence, and phase length, has been associated with changes in sporting workload, stress, and other behavioral variables (Shea and Vitzthum 2020). Therefore, sporting practitioners and female athletes often use menstrual cycle tracking applications, or athlete management systems (AMS), to record, track, and estimate menstrual cycle characteristics, including bleed onset, ovulation date, and menstrual cycle length in applied sports settings. Like menstrual cycle tracking apps, AMS software can be programmed to estimate athletes’ menstrual phases or their next bleed date, however, unlike automated apps, practitioners determine which mathematical equations to utilize, and are responsible for data interpretation. Several equations have been developed within the literature to predict the start of the next menstrual cycle and date of ovulation, enabling the cycle to be divided into estimated follicular and luteal phases at a minimum (McIntosh et al. 1980; Dupuit et al. 2023; Sohda et al. 2017). For example, Lamprecht and Grummer-Strawn (1996) developed an equation to estimate ovulation occurrence by taking the most recent menstrual cycle’s length and dividing it by two to get estimated ovulation. More recently, equations have become more complex, relying on retrospective regression-based models to generate ovulation date and next bleed estimates (Sodha et al., 2017; Soumpasis et al. 2020). However, from the equations published in the literature, there are inconsistencies in which are the most reliable, valid, and accurate at forecasting the next menstrual cycle bleed and ovulation date (McIntosh et al. 1980; Dupuit et al. 2023; Sohda et al. 2017).

It remains undisputed that the three-step best practice method (menstrual cycle tracking, urinary ovulation testing, and serum hormone analysis) is important for studying female participants in controlled research environments, however, this method is often impractical and unattainable in applied environments due to the requirement of frequent blood draws and ovulation testing (Schaumberg et al. 2017). Therefore, it is essential that methodologies are adapted to suit the practical environments they are collected in, yet still maintain scientific rigor (Elliot-Sale et al. 2025). As such, finding an accurate, reliable, and valid method for menstrual cycle tracking in the applied setting is an essential step towards comprehensively monitoring and forecasting the menstrual cycle and its characteristics in elite female athletes. Thus, a reliable approach would allow practitioners and athletes to be informed to make timely, proactive symptom interventions with greater confidence. Therefore, the aim of this study was to assess the reliability, accuracy, and validity of five mathematical equations throughout multiple cycles to predict menstrual cycle onset and date of ovulation in the applied environment.

## Methods

### Participants

Twenty-eight professional female soccer players (ages 18–42) from an elite National Women’s Soccer League (NWSL) team volunteered to take part in the study. Of the 28, only naturally menstruating or hormonal intrauterine device (IUD) users were included (*n* = 11, average ± SD: age: 27 ± 5 years; height 169.9 ± 8.9 cm; weight: 63.8 ± 7.3 kg). Of the 11, data from two participants were excluded from ovulation testing due to not having two positive ovulation tests, resulting in nine participants partaking in the ovulation portion of the study, where all participants (IUD and naturally menstruating) had two positive ovulation tests. Participants were considered naturally menstruating if they were not using any form of hormonal contraceptive, and had menstrual cycle lengths between 21 and 45 days. Written informed consent was obtained from participants prior to data collection and analysis. Ethical approval (study number 7862) was granted by Northumbria University Research Ethics Committee.

### Research design

Subjective and objective data were collected daily throughout the entire 2024 NWSL competitive season (275 days) as part of standard monitoring practices. Each morning participants completed a wellness questionnaire on a mobile platform, including adapted questions regarding female health (McNulty et al. 2024), which were input into five mathematical equations generated from current literature to inform the prediction of ovulation and next menstrual cycle bleed (Table 1). To establish the accuracy, reliability, and validity of the five equations at predicting day of ovulation, participants were asked to complete daily urinary ovulation testing from the last day of bleed until a positive test result was achieved, for two complete cycles. Within this paper, accuracy was defined as how close each equation predicted the start date of the next bleed and ovulation; reliability was defined as the ability to determine how consistent each equation was at predicting the start of each bleed and ovulation date across multiple cycles (within participants) and across participants (between participants); validity was chosen and defined as the extent to which predicted bleed and ovulation dates agreed with observed dates, reflecting the equations’ ability to accurately and reliably predict true menstrual and ovulation timing.

### Procedures

#### Menstrual cycle tracking

Prior to reporting to the training facility, participants filled out a daily wellness questionnaire to answer questions about their menstrual cycle including bleed report, day of cycle, symptoms experienced, and the severity of each symptom. The questionnaire took about one minute to complete every morning. From these data, menstrual cycle length and bleed length were established over 6–12 menstrual cycles, throughout the entirety of the 2024 NWSL season.

Starting on the day after their bleed ended, participants collected daily urine samples upon waking and used an over-the-counter ovulation prediction kit (Clear Blue, Swiss Precision Diagnostics, Switzerland) until a positive test result was achieved and confirmed with the lead researcher. Ovulation kits were used to detect a rise in luteinizing hormone, which increases 24 to 48-hours prior to ovulation, which was classified as 24 h post-positive ovulation test (Anderson et al. 2025). First day of bleed was used in the equations to predict the timing of ovulation within the next menstrual cycle. While data collection occurred across the entire season for the menstrual cycle length estimation aim, ovulation testing was only completed for two cycles to reduce player burden.

### Predictive equations

Table 1 provides the five equations that were used concurrently to predict ovulation and first day of the next bleed. The predicted dates generated from the equations were retrospectively compared to the actual dates of the criterion variables which were the first day of bleed (start of next cycle), and day of positive urinary ovulation test.

**Table 1:** Predictive equations, variables, and their assumptions generated from the literature

Equation	Equation Name	Formula	Variables Used	Assumptions
1	Ogino	Ovulation day *= (mcl – 15)*	Mean cycle length Bleed onset	Assumes a fixed length of a 14-day luteal phase and that ovulation occurs 15 days prior to the next day of bleed (Ogino, 1932).
2	Half Cycle Length	Ovulation day *= (mcl/2)*	Mean cycle length Bleed onset	Assumes that ovulation occurs at exactly halfway through the menstrual cycle, separating it evenly into the follicular and luteal phase (Lamprecht & Grummer-Strawn, 1996)
3	Optimized	Ovulation day = *0.501(mcl)−0.088* Luteal phase length = 0.466(mcl) + 0.088	Mean cycle length Bleed onset	Predicts the next ovulation date as a linear function of the relationship between the follicular phase length and mean length of the menstrual cycle (Sohda et al. 2017)
4	McIntosh	Follicular phase length = *0.767(rcl) – 7.556* Luteal phase length = *0.233(rcl) + 7.561*	Most recent cycle length Bleed onset	Divided into two parts to predict ovulation and cycle length by estimating follicular phase length, as well as luteal phase length. Ovulation is assumed to be the last day of the follicular phase (McIntosh et al. 1980)
5	Soumpasis	Ovulation day = *−5.2835 + (0.7344 x rcl*) Luteal phase = rcl -ovulation day	Most recent cycle length Bleed onset	Estimated the day of ovulation based upon the correlation between follicular phase and current cycle length (Soumpasis et al. 2020). Next bleed was estimated by subtracting the estimated ovulation from the recent cycle length to generate estimated luteal phase length.

*mcl (mean cycle length), *rcl* (most recent cycle length), mean cycle length calculated as a rolling average of previous minimum 3 + menstrual cycle lengths

### Statistical analysis

All numerical data are presented as mean ± SD. Significance was set at *p* < 0.05, and all data were analyzed using Microsoft Excel (Excel 2019, Microsoft Corporation, United States) and RStudio Core Team (R Foundation for Statistical Computing, Vienna, Austria). To evaluate the accuracy of each equation in predicting the next bleed and ovulation day, mean absolute error (MAE) was calculated and chosen due to its robustness to outliers, and ability to reflect the average absolute difference between predicted and actual dates in the same unit of measurement. Furthermore, a Bland-Altman analysis was run to demonstrate the agreement between predicted and actual dates, and used to assess systematic bias and limits of agreement. Intraclass correlation coefficients (ICCs) were used to assess the reliability of the predicted dates for both next bleed and ovulation day, measured across repeated cycles (6–12 cycles for bleed, and two cycles for ovulation), allowing for assessment of within subject reliability of each equation across multiple cycles. Absolute agreement was measured using a single-rater model ICC(3). The coefficient of variation (CV) was calculated to quantify cycle-to-cycle variability in both the actual (criterion) and predicted measures.

A linear mixed model (LMM) using the *lme4* package was chosen to determine if prediction error systematically differed by equation compared to the criterion (i.e. LH surge or day of bleed), to account for repeated cycles per participant, and to assess whether covariates and random effects influenced validity. Regression coefficients (β) were calculated to estimate the change in prediction error associated with each predictor variable, and each athlete was included as a random effect in the model. When a significant difference was identified, a post-hoc pairwise comparison of ovulation prediction was conducted using Tukey-adjusted estimated marginal means.

## Results

Eleven participants were included in the analysis; eight were naturally menstruating and three were IUD users. Equations are referenced in Table 1. Predicted bleed and ovulation estimates are shown in Table 2.

**Table 2:** Estimated bleed and ovulation days based upon equation, compared to actual bleed and ovulation days

Equation	Predicted Bleed Day	Actual Bleed Day	Mean Difference	MAE	Predicted Ovulation Day	Actual Ovulation Day	Mean Difference	MAE
**1**	26 ± 4	28 ± 5	−2	6	12 ± 4	17 ± 4	−5	5
**2**	27 ± 4	28 ± 5	−1	6	13 ± 2	17 ± 4	−4	4
**3**	26 ± 4	28 ± 5	−2	6	13 ± 2	17 ± 4	−4	4
**4**	27 ± 4	28 ± 5	−1	6	13 ± 3	17 ± 4	−4	4
**5**	27 ± 4	28 ± 5	−1	6	14 ± 3	17 ± 4	−3	3

*Mean ± SD* Data presented in days, *MAE* Mean absolute error

### Predicted versus actual day of the next menstrual bleed

**Fig. 1:**
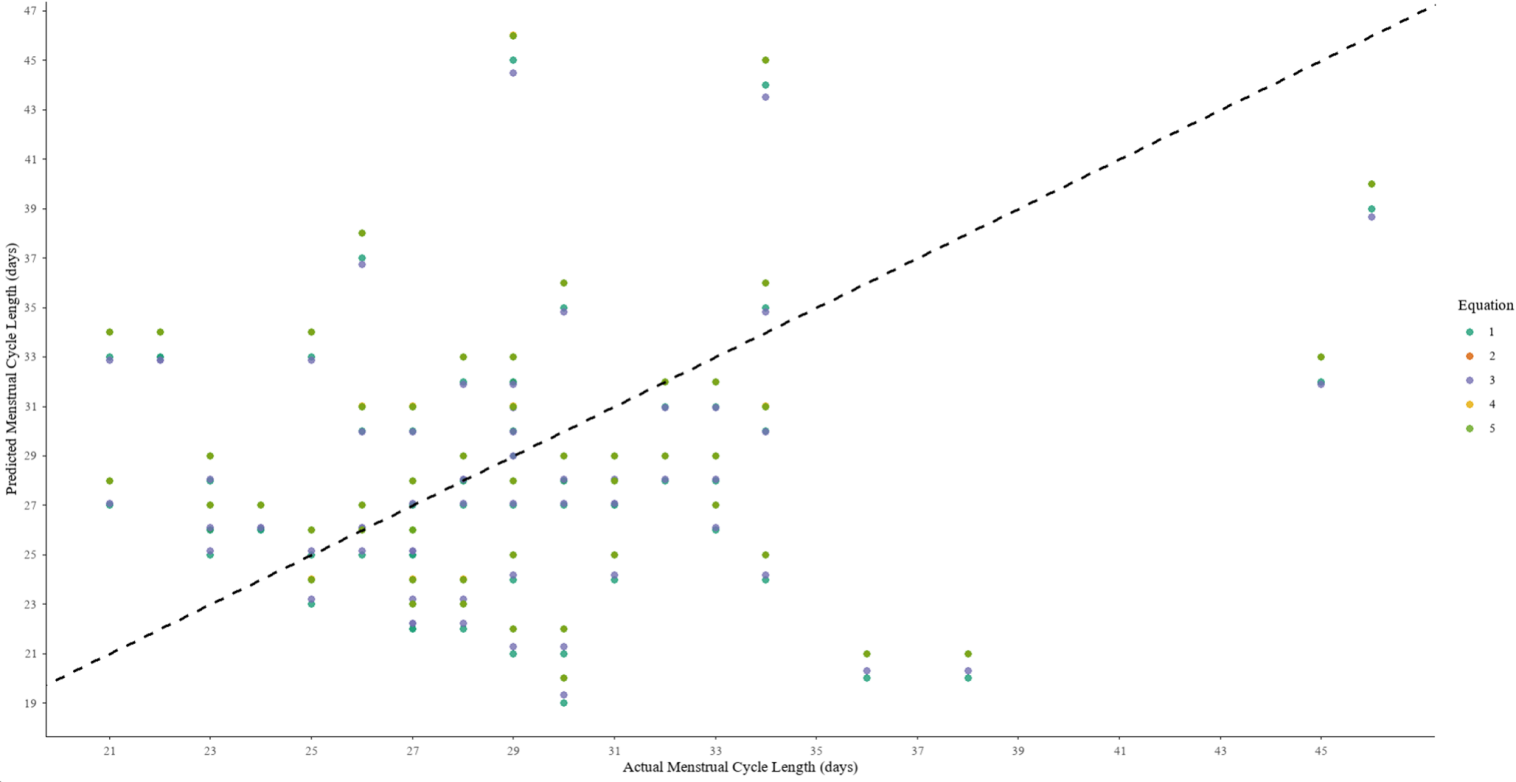
Accuracy of next bleed prediction for each equation, with estimations for every individual menstrual cycle. Dashed line is line of equality, which represents perfect agreement between predicted and actual values, where points above the line are overpredictions, and points below the line are underpredictions

There was no difference in the accuracy in predicting next bleed between all equations, where all equations had a MAE of 6 days (Table 2). Results of an ICC indicated moderate reliability across all five equations (ICC3 = 0.60, 95% CI [0.53–0.68], *p* < 0.001) (Fig. 1). Despite moderate reliability, there was high cycle to cycle variability in next bleed predictions (CV: 24.3 to 25.1%) indicating fluctuating precision between cycles. Results of a Bland-Altman analysis, including limits of agreement, are located in Table 3.

**Table 3:** Bias, standard deviation, and limits of agreements (LoA) in predicting next bleed

Equation	Bias	Standard Deviation	Lower LoA	Upper LoA
1	−0.859	6.41	−13.4	11.7
2	0.141	6.41	−12.4	12.7
3	−0.817	6.30	−13.2	11.5
4	0.146	6.41	−12.4	12.7
5	0.141	6.41	−12.4	12.7

Results of the robust LMM indicated there was no significant difference in prediction error between equations (β = −0.20, SE = 0.16, t(335) = − 1.22, 95% CI [− 0.65 − 0.24]). Additionally, hormonal profile did not significantly influence equation prediction error (β = −3.76, SE = 4.19, t(335) = −0.90, 95% CI [− 10.73–3.32]), though this was an exploratory aim due to the small sample size of IUD users. There was a significant positive association between cycle length and prediction error, with longer cycles associated with higher prediction error (β = 1.10, SE = 0.03, t(335) = 31.54, 95% CI [0.94–1.13]). Random intercepts were included for athlete (variance = 35.73, SD = 5.98), with residual variance of 17.97 (SD = 4.24), indicating substantial variance between participants (67%), and low within-athlete variance (33%), highlighting individual differences.

### Predicted versus actual day of ovulation

**Fig. 2:**
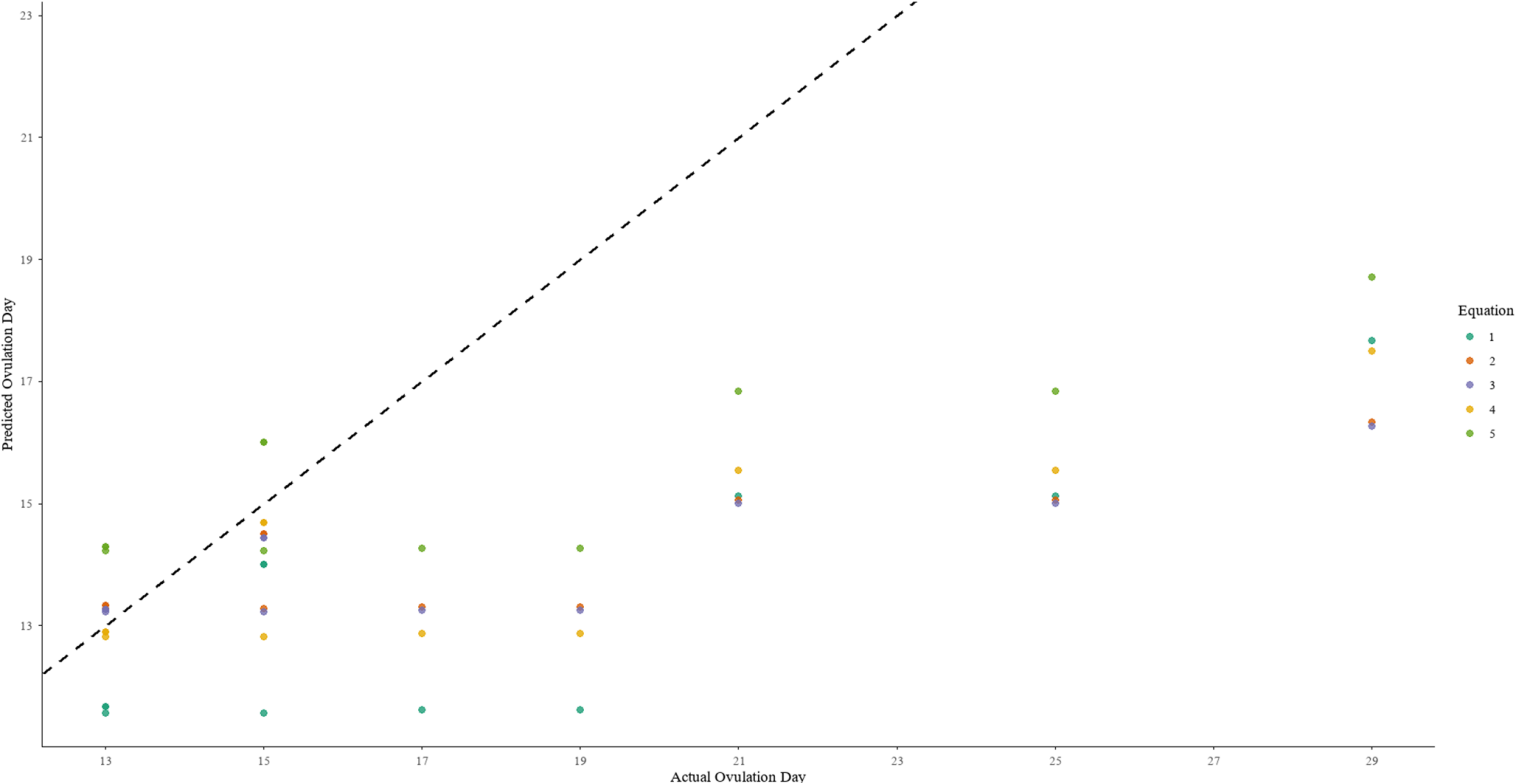
Accuracy of ovulation estimation prediction for each equation, with each individual menstrual cycle represented. Dashed line is line of equality, which represents perfect agreement between predicted and actual values, where points above the line are overpredictions, and points below the line are underpredictions

Predictive accuracy for ovulation was assessed using MAE, where Eq. 5 exhibited the lowest prediction error, however, there remained no difference in prediction error between all five equations (Table 2). The ICC3 indicated moderate reliability across all five equations (ICC3 = 0.45, 95% CI [0.18–0.75], *p* < 0.001) (Fig. 2). CV varied across equations, with Eq. 2 and Eq. 3 (8.50%) demonstrating the least relative variability, when compared with Eq. 1 (22.1%), 4 (16.5%), and 5 (14.6%), where precision was lower. Results of a Bland-Altman analysis, including limits of agreement, are in Table 4.

**Table 4:** Bias, standard deviation, and limits of agreements (LoA) in predicting ovulation

Equation	Bias	Standard Deviation	Lower LoA	Upper LoA
1	−5.25	6.32	−17.6	7.14
2	−3.62	5.06	−13.5	6.28
3	−3.69	5.06	−13.6	16.23
4	−4.04	5.67	−15.2	7.08
5	−2.64	5.59	−13.6	8.32

Results of the LMM indicated a significant difference in ovulation prediction error between equations, with Eq. 5 showing significantly lower prediction error than Eq. 1 (β = −1.15, SE = 0.54, t(44.98) = −2.14, *p* = 0.038). Results of a Tukey-adjusted estimated marginal means post-hoc analysis, however, revealed no statistically significant pairwise comparisons between equations. Random intercepts were included for athlete (variance = 21.6, SD = 4.67), with residual variance (1.59, SD = 1.26).

## Discussion

The present study examined the accuracy, reliability, and validity of five equations at predicting the start of the next menstrual cycle (bleed onset) and ovulation day in elite female soccer players. While findings of this study are preliminary due to a small sample size, the equations performed comparably when predicting the onset of the next bleed and ovulation, however, the precision of the equations decreased as menstrual cycle length increased, and thus, athletes and practitioners should be less reliant on equations when athletes have long or highly variable cycles. Furthermore, all equations had large variability in measurement and error rate, despite performing comparably. Thus, highlighting the importance of practitioners and athletes continuously monitoring menstrual cycle length and making adjustments to AMS when extended cycles are observed. While no single equation consistently outperformed the others in predicting the next menstrual bleed, Eq. 5 demonstrated the lowest error when predicting ovulation, and might offer practical utility for practitioners using and interpreting AMS, while also taking into account menstrual cycle characteristics reported from athletes.

### Predicting the start of the next menstrual cycle

Forecasting the next menstrual cycle bleed enables female athletes to be prepared for their next bleed and to employ active symptom mitigation plans (Chica-Latorre et al. 2025), which has been shown to improve menstrual cycle related discomfort, stress, and quality of life (Kennett et al. 2016). For some athletes, however, the biological variability in cycle length and inability to predict the start of the next bleed can cause additional stress (Chica-Latorre et al. 2025), further highlighting the importance of accurate menstrual cycle forecasting. In the present study, the difference between the equations predicting the next bleed was small and not significant. Interestingly, and similar to findings of several studies (Sohda et al. 2017; Soumpasis et al. 2020), there was a negative association between previous cycle length and prediction accuracy, indicating that longer cycles yielded higher prediction error among the five equations. Previous research suggests that predicting cycle length in advance of its commencement is difficult due to inherent intra-individual cycle variability (Dupuit et al. 2023; Soumpasis et al. 2020). Relatedly, researchers have found that fewer than 1% of participants had the same cycle length across four consecutive cycles, and over half showed fluctuations of five days or more (Soumpasis et al. 2020; Dupuit et al. 2023), similar to the prediction errors in the current study. Contrary to these findings, however, some studies have reported positive correlations between lengths of past and future cycles, meaning there remains a lack of consensus regarding next bleed and cycle length prediction (Creinin et al. 2004; Sohda et al. 2017). This highlights the need for practitioners to understand the increased error associated with extended cycles, in addition to understanding the cause, as well as the potential implications of extended menstrual cycles.

Understanding the impact of previous cycle length(s) is also important, as it underpins the premise of how each equation predicts the next bleed. For example, in an attempt to account for biological cycle variability, Eq. 3 uses the average of the previous cycle lengths, whereas Eqs. 4 and 5 are based upon the single preceding cycle length. Furthermore, Eq. 2 assumes that the follicular and luteal phases are equal in length, despite the follicular phase accounting for most of the biological variability in cycle length (Cole et al. 2009; Johnson et al. 2018) which might decrease predictive accuracy as cycle length increases (Sohda et al. 2017; Soumpasis et al. 2020). Despite these mathematical differences, no difference in prediction error was found between the five equations. As such, it remains plausible that monitoring menstrual cycle variability alongside AMS usage, and providing athletes with an estimated time-frame for bleed onset, even with some uncertainty, might still support proactive planning and reduce menstrual cycle-related anxiety or stress in the applied environment.

### Predicting day of ovulation

Ovulation estimation is commonly used as physiological marker, dividing the menstrual cycle into the follicular and luteal phase (Hackney 2016). The best practice methods, such as serum hormone analysis and ultrasonography (Mackens et al. 2017), are likely to be unrealistic in most applied environments. Alternatively, mathematical equations are often used to predict day of ovulation, yet there is no consensus on which one is the most valid or reliable (Dupuit et al. 2023; Soumpasis et al. 2020; Anderson et al. 2025). Results of the present study included high prediction error and no difference in the accuracy, reliability, or validity when predicting ovulation, with predicted day of ovulation occurring earlier than the positive ovulation test was reported. Johnson et al. (2018) reported that for applications or calendar counting methods based soley on cycle length, an eight-day estimated ovulation window has a 90% probability of including actual day of ovulation. All five equations within the present study predicted ovulation within a 3–4 day window, a slightly smaller window than Johnson et al. (2018) reported; a pattern that could potentially align with true biological variability in ovulation date. Sohda et al. (2017) reported that retrospective regression-based models are more valid than fixed-day calendar methods, which might explain why Eq. 5 had the lowest error. Due to the large measurement error in ovulation estimation, however, it remains unknown whether pinpointing the exact day of ovulation is realistic, especially when there is no clear consensus on the clinical definition (Maman et al. 2023). Undoubtedly, for clinical health insights (e.g., confirming eumenorrheic cycles) and research into the effect of ovarian hormone concentrations on an outcome (e.g., injury surveillance or performance), the three-step model proposed by Schaumberg et al. (2017) is imperative for reasons outlined by Elliott-Sale et al. (2025). Alongside longitudinal menstrual cycle data and practitioner interpretation in the applied setting, these equations might provide enough informed insight and athlete guidance when proactively mapping out day to day menstrual cycle symptoms and symptom mitigation strategies.

As with predicting day of bleed, there was large cycle to cycle measurement error when predicting ovulation date. While some biological variability is inherent and expected, ovarian cycle diversity must be further understood within elite athletes. In addition to tracking cycle characteristics, understanding biological variability is also essential as ovulatory disturbances have been associated with negative health markers, such as bone development and immunity, among others (Shea and Vitzthum 2020). It is recommended that practitioners utilize these equations to help inform their AMS, yet it is imperative that they consider and account for biological variability in ovulation when tracking and interpreting menstrual cycle characteristics. To help strengthen their predictions, practitioners might also consider incorporating objective measures such as basal body temperature, or subjective measures such as cervical mucus descriptions, as a feasible method to cross-reference the predicted day of ovulation within their AMS or monitoring systems (Ecochard et al. 2015; Su et al. 2017).

### Limitations

The authors of the current study recognize the difficulties of tracking menstrual cycles and mapping physical symptoms experienced across a menstrual cycle in the applied environment, however, this study is not without limitations. The study included a small sample size, especially for ovulation testing, yet it serves as a true reflection of elite team demographics. Given the small, elite-athlete sample size, these findings might not be generalizable to non-elite populations. Similarly, as this was an applied study, it was hard to control for external factors that might have affected the menstrual cycle and ovulation timing, such as training loads, energy intake, travel, outside stress, or potential underlying conditions (Shea and Vitzthum 2020; Anderson et al. 2025). Although the present study did verify an ovulatory window through LH surge, it is not the gold standard, or an exact marker of ovulation, and therefore might estimate it early or late. Ovulation verification, however, is often described as a barrier in applied based research, and thus, the gold-standard blood analysis for hormonal concentrations was not feasible, nor was it an aim of this study. Therefore, the conclusions of this study should not be applied to studies investigating the impact of hormonal concentrations across the cycle or to infer menstrual cycle status (e.g., eumenorrheic). Instead, the study provides a comprehensive evaluation of models that can be utilized within AMS or among practitioners to guide proactive symptom management based on estimated days of the cycle, while highlighting that practitioner understanding of biological cycle variability and the error associated with these equations is vital.

### Applied application

The results of this study helped to identify ways to predict the timeline for an athlete’s next bleed and ovulation, which might be used to inform a proactive symptom management plan alongside day-to-day symptom and severity monitoring, particularly within AMS. No equation emerged as superior, but based on being a regression model and displaying the highest accuracy among the five equations, Eq. 5 might be the most appropriate to use within AMS. However, the large measurement variability and error rate of the equations underscore the importance of using predictive models as general guides rather than exact predictors, which should be interpreted with caution, particularly among athletes with long (> 35 days) or highly variable cycles. A minimum of three cycles should be considered in a rolling average to account for biological variability that might naturally occur between cycles. When implementing into a real-world AMS system for the first time, previous cycle length(s) should be entered if known by the athlete, and if not, tracking should begin immediately with their next bleed, with practitioners exercising additional caution when analyzing and tracking the menstrual cycle until at least three full cycles’ data have been collected. Ovulation prediction is challenging and is often forecasted earlier than occurrence, and thus, highlights the importance of taking a multifaceted approach to menstrual cycle monitoring by utilizing additional indicators, such as ovulation testing, basal body temperature monitoring, or cervical fluid monitoring. As such, the ability to predict ovulation and timing of next bleed might help to inform athletes and practitioners of key time points, or estimated phases, throughout the cycle to help map out symptoms and symptom mitigation plans. While not precise, providing athletes with an estimated cycle forecast might also reduce cycle related stress, often experienced by those uninformed about their upcoming cycles.

## Conclusion

This was the first study to evaluate multiple predictive equations in an applied setting over several menstrual cycles. While no equation emerged as superior in terms of prediction accuracy, reliability, or validity, based upon these findings, data-informed prediction models outperformed calendar-based approaches, especially for ovulation prediction, yet, practitioners should exercise caution when cycle lengths are highly variable. There were no differences between the five equations in accuracy, reliability, and validity of predicting next bleed. There was evidence to suggest that Eq. 5 yields the highest accuracy at predicting ovulation, yet there was no difference in reliability and validity among the five equations when predicting ovulation. Results from this study provide athletes, practitioners, and researchers equations to concurrently map symptoms experienced across a menstrual cycle, and to help guide practitioners when providing symptom mitigation techniques based upon estimated days and time points within the cycle. Future research should focus on refining Eq. 5 (the Soumpasis method) to better predict the ovulatory timepoint and next bleed (Soumpasis et al. 2020). Furthermore, future research might examine the longitudinal variability among characteristics, such as bleed and cycle variability, as well as menstrual cycle symptom prevalence and severity to help provide athletes and practitioners tools to forecast and mitigate symptoms to allow athletes to perform at their best.

## Data Availability

Deidentified individual data that supports the results can be shared to the participants by request.

## Abbreviations

AMS: Athlete monitoring system
CV: Coefficient of variation
ICC: Intraclass correlation coefficient
IUD: Intrauterine device
LMM: Linear mixed model
MAE: Mean absolute error
MCL: Mean cycle length
NWSL: National women’s soccer league
RCL: Recent cycle length
